# Electrochemical Reduction of CO_2_ to Organic Acids by a Pd-MWNTs Gas-Diffusion Electrode in Aqueous Medium

**DOI:** 10.1155/2013/424617

**Published:** 2013-12-22

**Authors:** Guang Lu, Hui Wang, Zhaoyong Bian, Xin Liu

**Affiliations:** ^1^College of Environmental Science and Engineering, Beijing Forestry University, Beijing 100083, China; ^2^College of Water Sciences, Beijing Normal University, Beijing 100875, China

## Abstract

Pd-multiwalled carbon nanotubes (Pd-MWNTs) catalysts for the conversion of CO_2_ to organic acids were prepared by the ethylene glycol reduction and fully characterized by Fourier transform infrared spectroscopy (FTIR), X-ray diffraction (XRD), transmission electron microscope (TEM), X-ray photoelectron spectroscopy (XPS), and cyclic voltammetry (CV) technologies. The amorphous Pd particles with an average size of 5.7 nm were highly dispersed on the surface of carbon nanotubes. Functional groups of the MWNTs played a key role in the palladium deposition. The results indicated that Pd-MWNTs could transform CO_2_ into organic acid with high catalytic activity and CO_2_ could take part in the reduction reaction directly. Additionally, the electrochemical reduction of CO_2_ was investigated by a diaphragm electrolysis device, using a Pd-MWNTs gas-diffusion electrode as a cathode and a Ti/RuO_2_ net as an anode. The main products in present system were formic acid and acetic acid identified by ion chromatograph. The selectivity of the products could be achieved by reaction conditions changing. The optimum faraday efficiencies of formic and acetic acids formed on the Pd-MWNTs gas-diffusion electrode at 4 V electrode voltages under 1 atm CO_2_ were 34.5% and 52.3%, respectively.

## 1. Introduction

Recently, the conversion of CO_2_ to useful fuels as a carbon source has attracted much attention due to its greenhouse effect and fossil shortage [1]. Many attempts have been made to reduce the accumulation of atmosphere CO_2_, such as carbon capture and sequestration (CCS) [2], chemical usage [3], photochemical reduction [4], and electrochemical reduction [5]. In particular, electrochemical reduction can not only decrease CO_2_ release but also produce a variety of valuable compounds with high current efficiency. The products such as formic acid, CO, methane, ethylene, methanol, and ethanol can further be used as feedstock for chemical industry [6–9]. In addition, the electric power can be supplied by solar, wind, and geothermal energy, which are regarded as renewable sources [10]. It can also replace a part of fossil fuel consumption, which can further decrease the CO_2_ emission [6].

The research on electrochemical reduction of CO_2_ was first reported in the 19th century and rose since the energy crisis in the 1970's [11, 12]. Gas-diffusion electrodes could be operated at high current density (200–600 mA/cm^2^), that is, 10 folds higher than those achieved using metal plate electrodes for CO_2_ reduction [13]. Meanwhile, it improves the gaseous reactants distribution over the surface of catalyst. Catalysts with the unique three-phase interface structure lead to the least mass transport resistance across the gas-liquid interface and to the high catalyst surface. Supporting materials used to fabricate gas-diffusion, electrodes are mainly carbon materials, such as carbon fiber [14], carbon paper [15], carbon nanotubes [16–18], carbon clothes [19], and active carbon [20] owing to their resistance to acid/basic media, and possibility to control the porosity and surface chemistry within certain limits. Due to the nanometer size, low resistance, high surface area, chemical stability, special mechanical, and electronic properties, carbon nanotubes show excellent properties in microelectronics, composite materials, and electrical application. Hence, carbon nanotubes have been widely used for catalyst support as a suitable material [21–23]. Additionally, metal loaded carbon nanotubes (NTs) have been investigated extensively for the catalysis. Nanoscale metal catalysts could be dispersed on the high area of supported NTs. In the meantime, the interaction of carbon nanotubes with gas adsorbed either on or inside the tube or between aggregated MWNT attracts increasing attention due to the possible influence of the adsorption on some of the tubes properties and to the possibility of using these materials for efficient gas storage [24]. High concentrations of gas adsorbed by MWNTs can give rise to high-pressure-like effects at ambient pressures and promote the reduction by changing the reaction equilibrium. But until now, no sufficient investigation has been made on electrochemical reduction of CO_2_ on gas-diffusion electrode, using CNTs as support materials.

In this paper, Pd nanoparticle was decorated on MWNTs by ethylene glycol reduction. The prepared catalyst was then fabricated to the gas-diffusion cathode. The reduction of CO_2_ was performed in a cell with an organic synthetic diaphragm, a Ti/RuO_2_ anode, and self-made gas-diffusion cathode. On the base of catalyst and gas-diffusion electrode characterization, the key parameters of CO_2_ conversion were studied. Finally, the selectivity and efficiency of the formed products in the present and control systems were investigated.

## 2. Materials and Methods

### 2.1. Preparation of Pd-MWNTs Catalyst and Gas-Diffusion Cathode

The modifications of MWNTs (Shenzhen Nanotech Port Co. Ltd) were performed by heating 1.5 g MWNTs with 50 mL mixed concentrated acids (H_2_SO_4_-HNO_3_ mixture, 3 : 1 v/v ratio) for 1 h at 60°C. The product was filtered and washed with distilled water till neutrality. Then the solid was dried for 12 h at 70°C. Additionally 1.7 mg palladium chloride was dissolved in 15 mL ethylene glycol (reducing agent) with 13.6 mg sodium citrate to prevent the glomeration of palladium. After vigorously stirring for half an hour, ethylene glycol that contains 5% NaOH was added drop wise in order to maintain min pH at 10. Finally, treated MWNTs with the 100 : 1 mass ratio to palladium were added into the solution. After ultrasonic processing for 10 min, the vigorously stirred mixture was heated at 140°C. pH was kept above 10 by adding sodium hydroxide solution. The reaction was not over until the pH was constant. The mixture was then cooled down at room temperature, and filtered until no Cl^−^ was detected in filtrate. Remained solid was kept at 70°C for 12 h. Pd-MWNTs with a Pd load of 1 wt% were obtained. Gas diffusion layer was made of MWNTs. The gas-diffusion electrodes were made according to previous report [25]. It was made up of three parts: catalyst layer, gas-diffusion layer, and a stainless steel mesh. The catalyst layer was prepared with a mixture of 1.2 g 1 wt% Pd/MWNTs, 5.0 mL ethanol as a dispersant, and 3 g 10% PTFE as a wet-proofing agent and blinder. Beat and mix the mixture at 80°C until it emerged as a thin catalysts layer (5 cm × 6 cm, 0.4 mm thick). The gas-diffusion layer was prepared according to the procedure for making the catalyst layer except a mixture of 0.75 g of MWNTs, 0.05 g of acetylene black, 0.5 g of Na_2_SO_4_, 3.0 g of 10% PTFE, and 5.0 mL of ethanol was used. The stainless steel screen of 200 meshes as current collector was fist pretreated 0.5 h at 80–90°C by 1 L mixed alkaline solution that contained 20 g NaOH, Na_3_PO_4_, and Na_2_CO_3_ and then was pretreated 0.5 h by 0.5 mol/L hydrochloric acid solutions. Finally, catalyst layer, stainless steel screen, and gas-diffusion layer were cold pressed at 10 MPa for 1 min. The resulting electrode was then cut to 5 cm × 6 cm and about 0.4 mm thick.

### 2.2. Procedures

**Figure 1:**
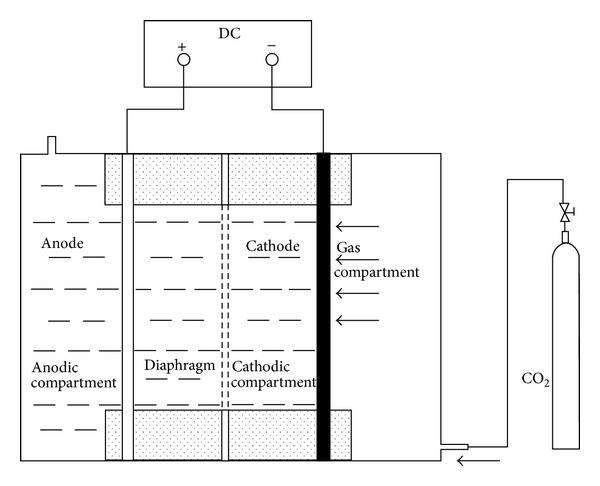
Schematic diagram of electrolysis apparatus.

Electrolysis was conducted in the terylene diaphragm cell of 100 mL. The anode was a Ti/RuO_2_ net (16 cm^2^) and cathode was a gas-diffusion electrode (16 cm^2^). A schematic of the experimental setup was shown in Figure 1. The electric power was provided by a laboratory direct current power supply with current-voltage monitor. CO_2_ was bubbled into the electrolyte for 30 min before the reaction. The gas-diffusion electrode was fed with CO_2_ continually during electrolysis. The distance between anode and cathode was 2 cm. Series concentrations of KHCO_3_ were chosen as electrolytes to investigate the suitable concentration of electrolytes. The reduction of CO_2_ was investigated in 0.1 mol/L, 0.3 mol/L, 0.5 mol/L, 0.8 mol/L, and 1.0 mol/L KHCO_3_ electrolyte at 25°C. The electrolysis voltages were 1 V, 2 V, 3 V, 4 V, and 5 V. During the preparative electrolysis, samples were taken (in 5 min periods) with a volume of 1 mL from the electrolyte in the middle of the reactor.

### 2.3. Analytical Methods

The XRD patterns were used to discern the identity of any phase present and their crystallite size. The Pd-MWNTs catalyst was characterized by XRD with a Rigaku D/max-III X-ray power diffractometer using Cu K*α* radiation with a Ni filter. The distribution of loaded palladium and catalyst morphology were obtained by transmission electron microscope (TEM) on a JEM-2010F transmission electron microscope (TEM) operated at an accelerating voltage of 200 kV. The surface composition of catalyst and Pd surface concentration were then investigated by XPS on PHI5300 Electron Spectrometer (Mg Ka radiation; hv = 1253.6 eV). XPS data were calibrated using the binding energy of C1s (284.6 eV) as the standard. Fourier transform infrared spectroscopy (FTIR) was used to analyses change of functional groups of MWNTs by Excalibur 3100. Spectra were obtained in an optical range of 1000–2000 cm^−1^ at a resolution of 0.2 cm^−1^. The cyclic voltammetry (CV) was recorded using a potentiostat/galvanostat (EG&G Model 273A) with a standard three-compartment cell consisting of a Pt wire as a counter electrode, a Ag/AgCl electrode as a reference electrode, and the Pd/MWNTs catalyst modified electrode as a working electrode. The Pd/MWNTs catalyst modified electrodes were made according to previous report [26]. 0.5 mol/L KHCO_3_ solutions were used as electrolytes, which were saturated with CO_2_ (N_2_) to the cell for 30 min before the electrochemical measurements, and gas was continued throughout the electrolysis. The temperature was kept constant at 25°C during the experiment. The scanning rate was 100 mV/s. Soluble products were determined using an ion chromatograph (DIONEX ICS3000) analyses by comparing the retention time of the standard reference compounds. The separation was performed using an AS-11 column at the flow rate of 1.2 mL/min with 250 mmol/L NaOH as the mobile phase at 30°C. The total run time was 15 min and an injection volume is 10 *μ*L.

## 3. Results and Discussion

### 3.1. Characterization of Pd-MWNTs Catalyst

#### 3.1.1. FTIR of Pretreatment MWNTs

**Figure 2:**
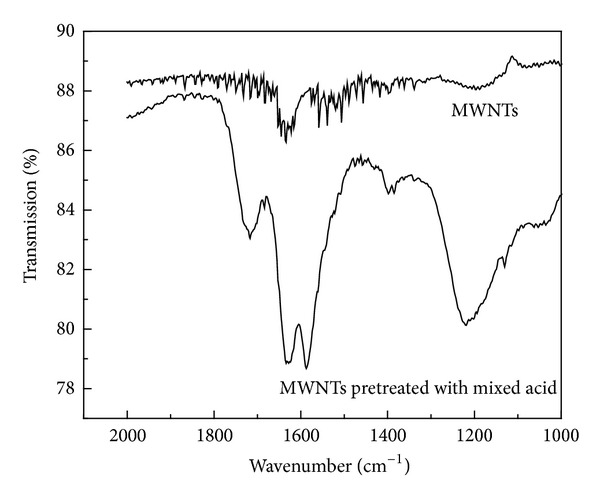
FTIR pattern of MWNTs and MWNTs pretreated with mixed acid.

To disperse metal catalysts on the surface of MWNTs, the modification of MWNTs that could induce the chemical reaction reactive sites must be done first. Mixed acid oxidization of NTs was used to produce functional groups. The typical FTIR spectra of MWNTs and MWNTs pretreated with mixed acid are showed in Figure 2. The MWNTs had two characteristic infrared absorption peaks at 1625 cm^−1^ and 1575 cm^−1^. The peak at 1575 cm^−1^ corresponded to the E1u vibrational modes of carbon nanotubes wall that indicated the presence of graphite structure of MWNTs. The peak at 1384 cm^−1^ originated from the O–H bending vibration of –COOH, but the peak shape was not very clear, indicating that there were few carboxyl groups on the surface of untreated MWNTs. As showed in the FTIR spectra of MWNTs pretreated with mixed acid, the introduction of −COOH could be observed from the peak 1732 cm^−1^ that corresponded to the –C=O stretching frequencies. These functional groups played a key role in depositing palladium on the surface of MWNTs and their intensities were greatly enhanced after mixed functionalization [26]. There were electrostatic adsorptions or coordination effect between palladium chloride and functional groups on the surface of MWNTs. Then palladium chloride was reduced by ethylene glycol and the palladium nanoparticles were formed on the surface of MWNTs in situ.

#### 3.1.2. XRD and TEM Analysis of Pd-MWNTs Catalyst

**Figure 3:**
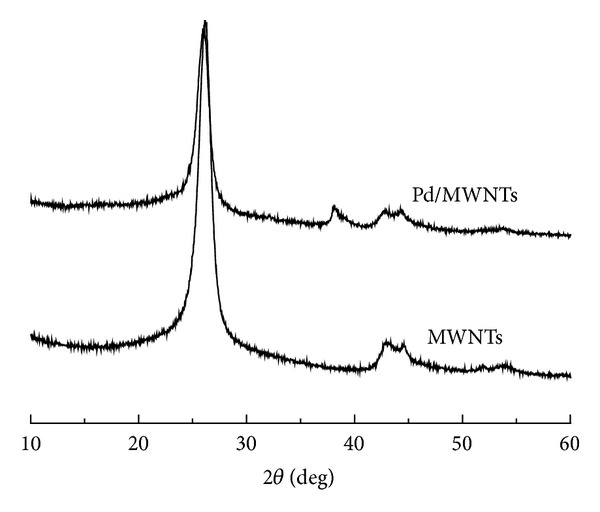
XRD pattern of MWNTs and Pd-MWNTs catalysts.

The XRD patterns of Pd-MWNTs catalysts are shown in Figure 3. The main characteristic peak emerged at 39.2° corresponding to the Pd particle, which indicated the successful reduction of metal salt [27]. The Pd peak became narrower and sharper with the increasing diameter of Pd particles. XRD analysis showed that there was no detectable crystal structure of Pd particles over the 1 wt.% Pd-MWNTs catalyst. This result indicated that Pd particles in 1 wt. % Pd-MWNTs were amorphous. The diffraction peaks at 26°, 42.8°, and 54.5° observed in the diffraction of MWNTs could be attributed to the hexagonal graphite structures (002), (100), and (004) [28], respectively, which demonstrated that modified MWNTs still had a high electrical conductivity as original one after oxidation and reduction and thus could be good supporting materials.

**Figure 4:**
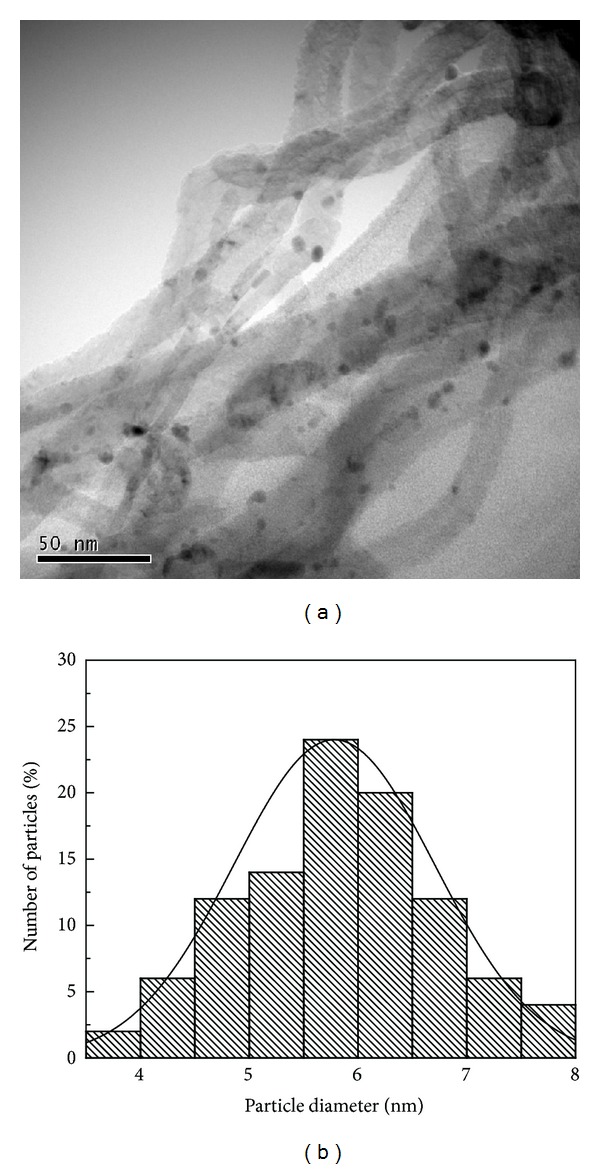
TEM pattern and the particle size of Pd-MWNTs.

Figure 4 presented a TEM image of MWNTs that were covered with Pd nanoparticles. Together with the XRD pattern, these observations suggested that Pd nanoparticles were amorphous and highly dispersed over MWNTs. There was no obvious aggregation on the image. The average particle size was 5.7 nm. A lot of Pd particles were formed in the port and bend part of MWNTs, where defective sites and foundation groups were easily formed after nitro-sulfuric acid treatment.

#### 3.1.3. Electrochemical Analysis of Pd-MWNTs Catalyst

**Figure 5:**
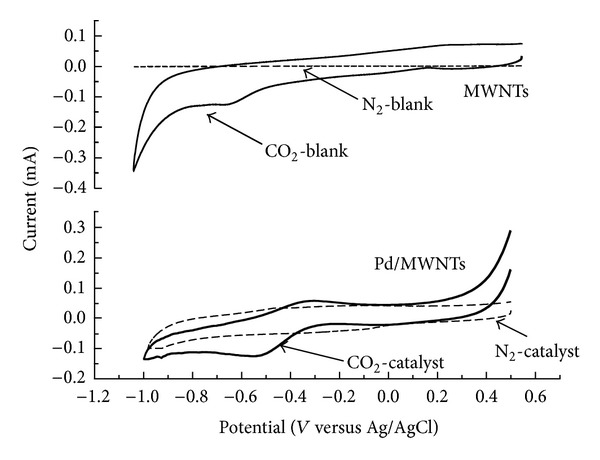
Cyclic voltammograms for MWNTs and Pd-MWNTs by catalyst modified glassy carbon electrode in 0.5 mol/L KHCO_3_ solution.

In order to elucidate the catalytic function of Pd-MWNTs composites, cyclic voltammogram for MWNTs and Pd-MWNTs by catalyst modified glassy carbon electrode in 0.5 mol/L KHCO_3_ solution are shown in Figure 5.

As shown in Figure 5, the cathodes currents obtained under 1 atm N_2_ were suppressed compared with those obtained under 1 atm CO_2_. No significant responses beyond the background current were observed for MWNTs and Pd-MWNTs modified glassy carbon electrode under N_2_ purging condition. Under CO_2_ purging condition, MWNTs and Pd-MWNTs modified glassy carbon electrode showed reduction peaks at ca. −0.65 V and −0.52 V, respectively. These results indicated that CO_2_ could take part in the reduction reaction directly. It could be inferred from the fact that CO_2_ was one key point of the reaction. By bubbling gas through the electrolyte and checking out the change of partial current density resulted from products formation, Hori [29] found that HCO_3_^−^ was not an electroactive species, assuming that HCO_3_^−^ was first decomposed to CO_2_ and CO_2_ was reduced at the electrode afterwards. The whole reaction process was as follows: CO_2_ was converted into HCO_3_^−^ first and then was transport to the surface of palladium nanoparticles in the form of HCO_3_^−^; HCO_3_^−^ was decomposed to CO_2_ and CO_2_ was reduced at the electrode at last. The CO_2_ could be chemisorbed as a bent CO_2_^*δ*−^. Pd has been shown to strongly adsorb CO_2_ [30]. The electrode metals interacted with carbon or oxygen coordination or mixed coordination adsorption mode, respectively. It is well known that the decomposition rate of HCO_3_^−^ is slow in ambient temperature and pressure, so the decompose of HCO_3_^−^ was the rate determining step in the reaction. Moreover, the reduction current peak of the Pd-MWNTs modified electrode was more positive and higher than that of MWNTs modified electrode under 1 atm CO_2_. It indicated that Pd-MWNTs catalysts showed high catalytic performance in the electrochemical reduction of CO_2_.

### 3.2. Electrochemical Reduction of CO_**2**_ to Organic Acids at Pd-MWNTs Gas-Diffusion Electrodes

The influences of electrolysis voltage, reaction time, and electrolyte concentration were discussed, and relations between reaction conditions and products formed were presented. The products distributions and influencing factors were discussed blow.

#### 3.2.1. Role of Electrolyte Concentration

**Figure 6:**
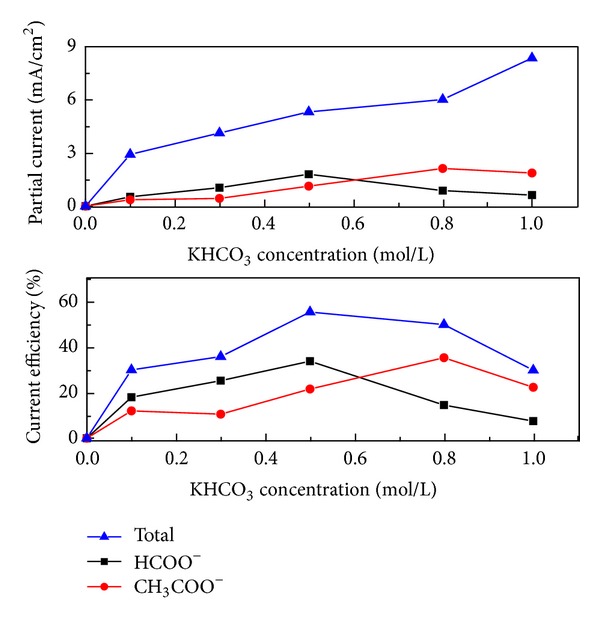
Variation of formic acid and acetic acid via KHCO_3_ concentration (4 V, 20 min).

Most of the works were carried out in the aqueous electrolyte of KHCO_3_, but the concentrations were different, ranged from 0.1 to 0.5 mol/L [10, 16]. The electrode voltage was 4 V and electrolysis time was 20 min.  Figure 6 shows the correlation between the KHCO_3_ concentrations, partial current, and current efficiency at the Pd-MWNTs gas-diffusion electrode for the reductions of CO_2_. Formic acid and acetic acid were the main products.

As shown in Figure 6, high concentration of electrolytes enhanced the conductivity of the solution. The current densities of formic acid and acetic acid increased with the increasing electrolyte in a certain range. The current density of formic acid reached up to 1.8 mA/cm^2^ in the 0.5 mol/L KHCO_3_ electrolytes. The highest current density of acetic acid was 2.13 mA/cm^2^, which was obtained from 0.8 mol/L KHCO_3_ solutions. The same trends have taken place in current efficiencies of formic acid (33.8%) and acetic acid (35.3%). The total current efficiency of 0.5 mol L^−1^ KHCO_3_ electrolyte 55.4% was the highest, followed by 0.8 mol/L KHCO_3_ electrolytes 49.9%.

The CO_2_ reduction was greatly limited by the low solubility and mass transfer of CO_2_ [31]. The kinetic control was likely to be related to the release of free molecular CO_2_ from aqueous bicarbonate. The reduction was much affected by decomposition of HCO_3_^−^. By using the gas diffusion electrodes, a great amount CO_2_ was bubbled into the electrolyte and reacted with OH^−^. Then CO_2_ could be carried to the cathode surface more easily in the water-solution ionic solution than across the gas-liquid interface to catalyst surface. The CO_2_ was chemisorbed by Pd and then reduced to formic acid and acetic acid [30]. Hence, the gas-diffusion electrode increased the reaction rate.

The pH at the electrode was also greatly affected by the electrolyte, since OH^−^ was released in the electrode reactions. Hori [29] found that product selectivity depends upon availability of hydrogen or protons on the surface, which was controlled by pH at the electrode. In this experiment, KHCO_3_ electrolyte with somewhat buffer ability that can neutralize OH^−^ and CO_2_ also acted as both a react ant and a buffer. High pH induced negative electrocatalytic potential for CO_2_ reduction, which decreased the efficiency of formic acid and acetic acid formation [32]. While high concentration of HCO_3_^−^ can keep the pH of electrolyte steady, low pH promoted hydrogen evolution in the mean time. Hydrogen evolution reaction also went up with KHCO_3_ concentration. Hence the current efficiencies of formic acid and acetic acid decreased when high concentration electrolyte was employed.

#### 3.2.2. Influence of Voltage

**Figure 7:**
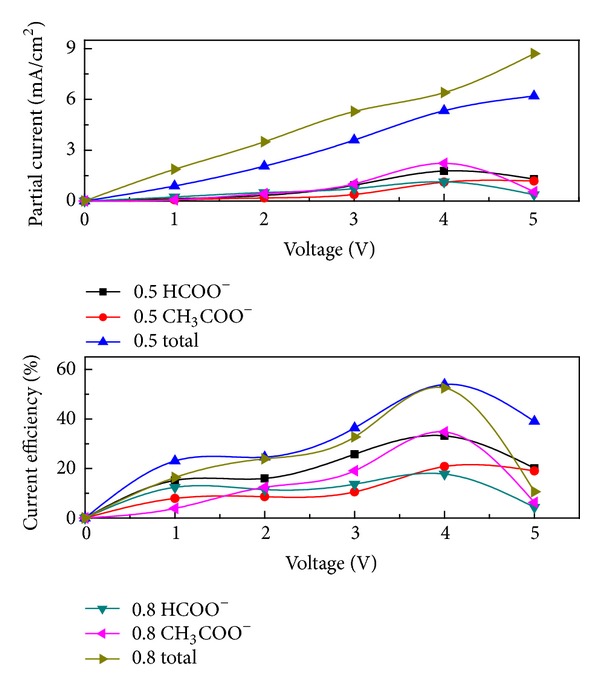
Correlation between the voltage, partial current density, and current efficiency of reduction products formed in the electrochemical reduction in 0.5 mol/L and 0.8 mol/L KHCO_3_ solutions (20 min).

The voltage and concentration of CO_2_ reduction products at gas-diffusion electrodes are plotted against the electrode voltage in Figure 7. The formations of formic acid and acetic acid were detected in 0.5 mol/L and 0.8 mol/L KHCO_3_ solutions, respectively.

As shown in Figure 7, the total current densities gradually increased with the electrode voltages. The total current efficiencies, current densities, and efficiencies of formic acid and acetic acid reached the maximum at 4 V. At this point the maximum current efficiency of formic acid was 33.2%; the maximum current efficiency of acetic acid was 34.8%. The total current efficiencies in 0.5 mol/L and 0.8 mol/L KHCO_3_ solutions were 54% and 52.6%, respectively. Hence 4 V of electrode voltage was chosen as the optimal voltage for the electrolysis under present conditions.

The reduction of CO_2_ was a complex multistep reaction [29]. CO_2_ reduction did not occur easily and the applied electrolysis potentials for CO_2_ reduction were more negative than the equilibrium value because the single electron reduction of CO_2_ to CO_2_^•−^, which was recognized as the first step to activate CO_2_ for subsequent reduction steps, occurred at −1.90 V versus NHE due to a large reorganizational energy between liner molecule and bent radical anion. This step has also been determined as the rate determining step for CO_2_ reduction. As shown in Figure 7, the current densities of formic acid and acetic acid began to rise quickly near 3 V. A large number of CO_2_^•−^ supply became possible when high voltage was applied.

When the voltage was low, the reaction was controlled by electron transfer obviously. Therefore, with the increase of electrode voltage, the production of formic acid and acetic acid also increased. The formations of formic acid and acetic acid were still controlled by electron transfer when high voltage was employed. The gas-diffusion electrode feeding CO_2_ to the electrocatalyst contributed to the high electrolysis efficiency [33]. When the voltage was high, hydrogen also formed on the cathode. Hence, further increase of voltage did not increase the current efficiency of organic acid.

#### 3.2.3. Study on Reaction Time

**Figure 8:**
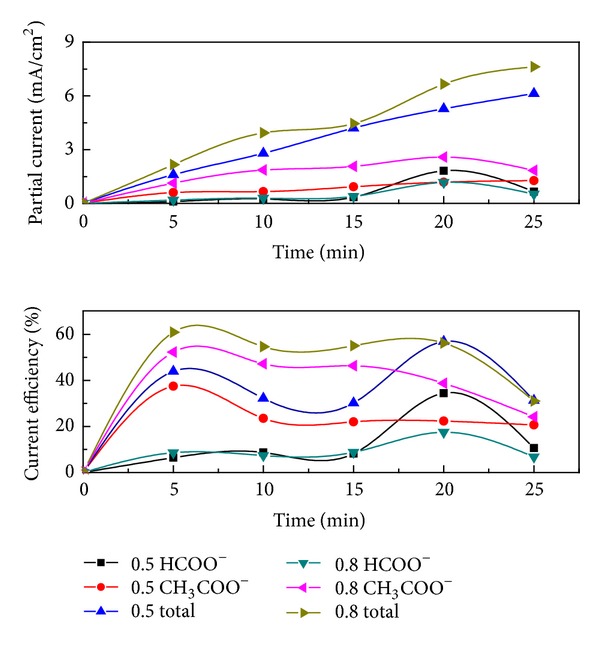
Variation of formic acid and acetic acid via time (4 V, 0.5 mol/L and 0.8 mol/L KHCO_3_ solutions).

Figure 8 shows the concentration changes of the formed formic acid and acetic acid with the reaction time.

As shown in Figure 8, the current efficiency for formic acid formation increased with increasing the time and reached a maximum of 34.5% at 20 min in 0.5 mol/L KHCO_3_ electrolytes; the current efficiency for acetic acid formation reached a maximum of 52.3% at 5 min in 0.8 mol/L KHCO_3_ electrolytes.

It is easier to generate acetic acid as the multielectron transfer reaction of CO_2_ reduction is thermodynamically conducive to single electron transfer reaction [29]. The numbers of electrons participated in the reaction have a great influence on the product selectivity. When the supply of electron was relatively abundant both in 0.5 mol/L and 0.8 mol/L KHCO_3_ electrolytes, the formation of acetic is the primary reaction. As shown in Figures 6 and 7, when the voltage was low or the concentration of electrolyte was light, the supply of electron was insufficient. Then, the current efficiency of formic acid is high and its formation was the primary reaction. The current efficiencies declined when the electrolysis time exceeds 20 min. Further electrolysis induced the decomposition of formic acid and acetic acid which competed with the CO_2_ reduction.

To check the steady of Pd-MWNTs catalysts gas-diffusion electrode, three recycled batches of experiment results were listed below under the same condition (0.5 and 0.8 mol/L KHCO_3_, 20 min, and 4 V) in Table 1.

The faraday efficiencies of products in 0.5 mol/L KHCO_3_ electrolytes almost remained unchanged. However the faraday efficiencies of products in 0.8 mol/L KHCO_3_ electrolytes changed slightly due to complexity caused by high concentration electrolyte. Results indicated that the catalyst remains as high activity and reaction systems keep their steady.

It can be concluded that electrolyte concentration, voltage, and reaction time had considerable influence on the species and amount of the products. High voltage and high concentration electrolyte advanced the formation of acetic acid. The best reaction conditions for the electrochemical reduction of CO_2_ to acetic acid were 4 V, 5 min and 0.8 mol/L KHCO_3_. The current efficiencies of acetic acid and formic acid were 52.3% and 8.6%, respectively and the total current efficiency was 60.9%. The best reaction conditions for the electrochemical reduction of CO_2_ to formic acid were 4 V, 20 min and 0.5 mol/L KHCO_3_. The current efficiencies of formic acid and acetic acid were 34.5% and 22.4%, respectively; the total current efficiency was is 56.8%.

### 3.3. Catalysis Analysis of Pd-MWNTs Gas-Diffusion Electrode

**Figure 9:**
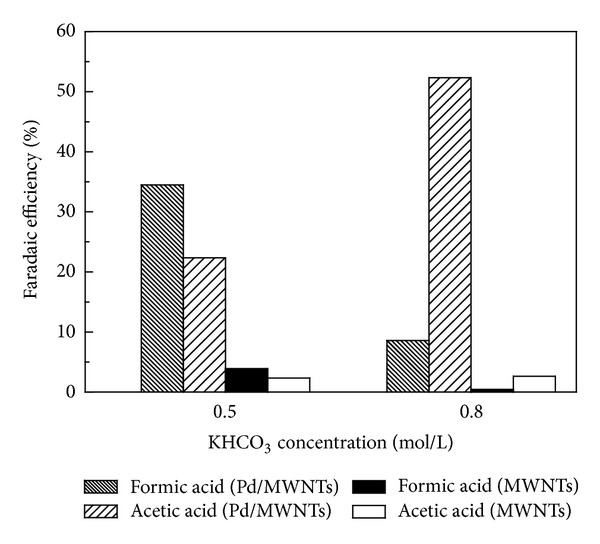
Comparison of faradaic efficiency depended for the products in 0.5 mol/L KHCO_3_ (4 V, 20 min) and 0.8 mol/L KHCO_3_ (4 V, 5 min) by electrochemical reduction of CO_2_.

Comparisons of faraday efficiencies for the products by electrochemical reduction of CO_2_ on the MWNTs and Pd-MWNTs gas-diffusion electrode systems are shown in Figure 9.

The electrochemical reduction of CO_2_ in the Pd-MWNTs gas-diffusion electrode system resulted in a maximum formation of acetic acid with a faraday efficiency of 52.3% and minimum formic acid 8.6% in 0.8 mol/L KHCO_3_ at 4 V. Acetic acid and formic acid were produced at faradaic efficiency of 22.4% and 34.5%, respectively, in 0.5 mol/L KHCO_3_ at 4 V. High efficiency and selectivity were achieved when Pd-MWNTs gas-diffusion electrode was employed. The Pd-MWNTs gas-diffusion electrode had high catalytic activity in the reduction of CO_2_ under the optimal conditions compared with that without Pd nanoparticles. High efficiency attributed to stronger interaction between Pd and MWNTs support. Additionally, Pd catalysts play an important role in the current efficiency and product selectivity.

## 4. Conclusions

A Pd-MWNTs gas-diffusion electrode was prepared for the electroreduction of CO_2_. In the fully characterized catalysts, Pd particles of an average size of 5.7 nm were highly dispersed in carbon nanotubes with amorphous structure. The CV behaviors showed that the Pd-MWNTs catalysts promoted the reduction by lowering the reduction potential and increasing the reduction current. In addition, electrochemical reduction of CO_2_ at Pd-MWNTs gas-diffusion electrodes was investigated in a terylene diaphragm cell. 4 V of electrode voltage was the optimal voltage for the present system. The suitable KHCO_3_ electrolyte concentrations for the formic acid and acetic acid were 0.5 mol/L and 0.8 mol/L, respectively. The optimum faraday efficiencies of formic and acetic acids formed on the Pd-MWNTs gas-diffusion electrode at 4 V electrode voltages were 34.5% and 52.3%, respectively.

## Figures and Tables

**Table 1:** Test of steady of Pd-MWNTs catalysts gas-diffusion electrode.

Electrolyte concentration (mol/L )	Faradaic efficiency (%)			
	HCOOH	CH_3_COOH	Total	Average total
0.5 mol/L KHCO_3_	33.8	21.6	55.4	55.4
	33.2	20.8	54.0	
	34.5	22.4	56.8	

0.8 mol/L KHCO_3_	14.7	35.3	49.9	52.9
	17.8	34.8	52.6	
	17.5	38.7	56.2	
